# Optimization of Near-Infrared Fluorescence Voltage-Sensitive Dye Imaging for Neuronal Activity Monitoring in the Rodent Brain

**DOI:** 10.3389/fnins.2021.742405

**Published:** 2021-10-27

**Authors:** Rebecca W. Pak, Jeeun Kang, Emad Boctor, Jin U. Kang

**Affiliations:** ^1^Department of Biomedical Engineering, Johns Hopkins University, Baltimore, MD, United States; ^2^Department of Radiology and Radiological Science, Johns Hopkins University, Baltimore, MD, United States; ^3^Department of Electrical and Computer Engineering, Johns Hopkins University, Baltimore, MD, United States

**Keywords:** voltage-sensitive dye, fluorescence imaging, brain activity, near-infrared, dosage optimization

## Abstract

Many currently employed clinical brain functional imaging technologies rely on indirect measures of activity such as hemodynamics resulting in low temporal and spatial resolutions. To improve upon this, optical systems were developed in conjunction with methods to deliver near-IR voltage-sensitive dye (VSD) to provide activity-dependent optical contrast to establish a clinical tool to facilitate direct monitoring of neuron depolarization through the intact skull. Following the previously developed VSD delivery protocol through the blood-brain barrier, IR-780 perchlorate VSD concentrations in the brain were varied and stimulus-evoked responses were observed. In this paper, a range of optimal VSD tissue concentrations was established that maximized fluorescence fractional change for detection of membrane potential responses to external stimuli through a series of phantom, *in vitro*, *ex vivo*, and *in vivo* experiments in mouse models.

## Introduction

It is well-established that neuron activity is the basis for brain function and more generally, organism behavior. Many tools have been developed to study the complex nature of how the brain works. Some of these techniques rely on bulk physiological effects, such as the blood-oxygen-level-dependent (BOLD) signal which utilizes hemodynamics as an indicator of neuron activity. This proxy may be valid in the healthy brain but is often compromised in the face of diseases that can cause neurovascular uncoupling among other biological alterations (Pak et al., 2017). There have also been profound investigations using photoacoustic and ultrasound as alternative modalities, but their contrast remained limited by neurovascular physiology such as oxyhemoglobin saturation and cerebral blood volume or flow (CBV or CBF) (Hu and Wang, 2010; Errico et al., 2015; Kang et al., 2018). Although methods such as electroencephalography have also been used for direct monitoring of electrical activity, these methods tradeoff spatial resolution (Burle et al., 2015).

Optical imaging is well-suited to address these drawbacks due to its high spatial and temporal resolutions that allow visualization of individual cells as well as populations on millisecond time scales. However, neurons and glia lack natural activity-dependent optical contrast (Kim and Jun, 2013). Most often, this contrast is added in the form of fluorescent molecule tags (Shimomura, 1979; Tsien, 1989, 2002; Chalfie et al., 1994). In neuroscience applications, transgenic animal models have been employed to observe calcium signaling (Tian et al., 2009; Madisen et al., 2010; Zariwala et al., 2012; DeNardo and Luo, 2017). Yet, to make a clinically relevant protocol, genetic modification and the accompanying skull thinning or removal surgeries for visible-wavelength imaging should be avoided.

Hence, injectable near-IR voltage-sensitive dyes (VSDs) that can be imaged through the skull were investigated as an alternative that facilitates the possibility of translation into human medical practice. We recently demonstrated a transmembrane VSD redistribution mechanism in functional photoacoustic imaging (Kang et al., 2019, 2020b), but its interherent dual-modal contrast in fluorescence has not been fully explored. The VSD administration was designed in a heuristic way, rather than providing quantitative guidelines to secure both high functional contrast, with maximized signal intensity changes between resting and stimulated states, and imaging sensitivity in the rodent brain. In this paper, we optimized *in vivo* VSD protocols in mice to maximize sensitivity and functional contrast in near-infrared fluorescence VSD imaging to provide guidelines for synergetic research in our dual-modal, minimally invasive neuroimaging using fluorescence and photoacoustics. An extensive range of studies was designed and performed using phantoms, *in vitro* cultures, and *ex vivo* tissues. A range of optimal VSD tissue concentrations was defined in order to avoid natural fluorescence quenching effects due to the formation of VSD aggregates in the extracellular space while maximizing the signal sensitivity and functional contrast in fluorescence VSD imaging. The investigation was supported by a versatile wide-field fluorescence imaging system for both cellular and tissue-level recordings.

## Materials and Methods

### Dye Selection and Mechanism of Action

**FIGURE 1:**
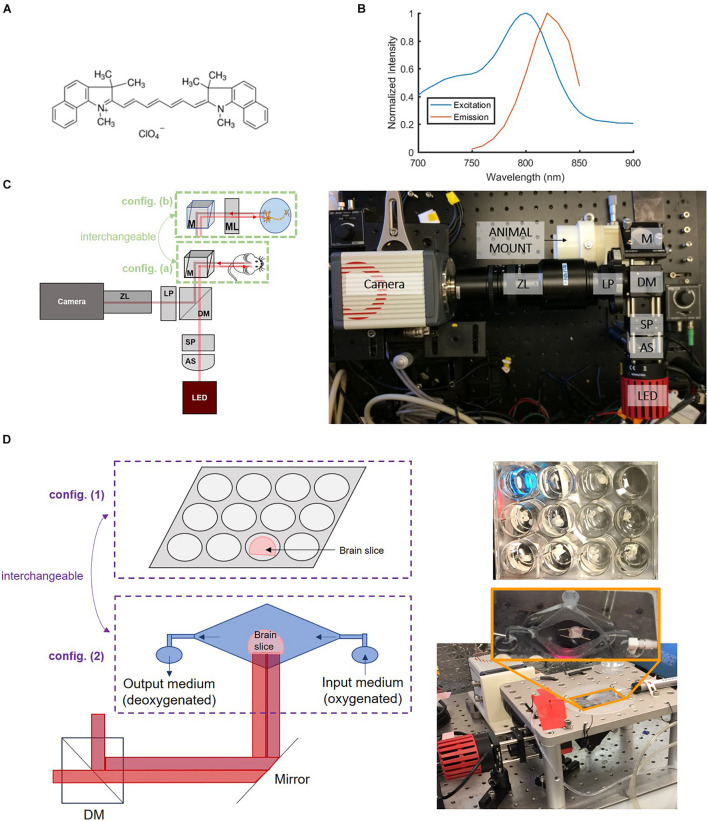
Near-IR VSD IR-780 perchlorate’s **(A)** chemical structure and **(B)** excitation-emission characteristics collected by spectrophotometry, which informed the **(C)** design of the versatile wide-field fluorescence imaging system with two configurations: config. (a) for larger field of view and config. (b) for greater magnification to achieve cellular resolution for *in vitro* experiments. **B**: Adapted with permission from Pak et al. (2018) © The Optical Society. AS = aspheric lens, SP = shortpass filter, DM = dichroic mirror, LP = longpass filter, ZL = zoom lens, M = mirror, ML = magnifying lens. **(D)** Samples were also monitored in two configurations for *ex vivo* brain slice studies: config. (1) a well plate setup and config. (2) a “bath” with continuous flow of new oxygenated medium to promote improved cell viability.

IR-780 perchlorate VSD was chosen for its excitation-emission characteristics which, unlike many common VSDs (Fluhler et al., 1985; Petersen et al., 2003; Baker et al., 2005; Robinson et al., 2011; Woodford et al., 2015), operates in the near-IR region. The use of near-IR wavelengths decreased absorptive and scattering losses and led to increased penetration depth, which facilitated through-skull imaging. IR-780 perchlorate’s mechanism of action, like other cyanine voltage-sensitive dyes, is based on the aggregation of VSD molecules that reflect the electrical state of neurons. Its chemical structure (Figure 1A) with systems of delocalized electrons is responsible for its near-IR excitation-emission spectra (Figure 1B). It is also a positively charged molecule which leads to its voltage sensitivity, manifested by the movement of these molecules into and out of the cell membrane in response to charge gradients. The fluorescence signal intensity is modulated by variations in local concentrations of VSD molecules. High levels of VSD aggregation disrupt fluorescence, leading to low intensity signals. Dye molecules go into cells at resting (or negative) membrane potential due to their positive charge. Since a single cell contains a relatively small volume as compared to the extracellular matrix, this makes the local concentration of VSD high. At high concentrations, VSD molecules interact with each other and aggregate which degrades and quenches the fluorescence. When action potentials occur and the membrane is depolarized, VSD aggregates disperse by lack of neuronal attraction, effectively creating a low concentration environment where VSD molecules do not interact with each other, leading to high fluorescence intensity. Thus, the flow of VSD molecules into and out of cells due to membrane potential changes allows cellular activity to be monitored through fluorescence imaging.

The VSD mechanism based on transmembrane redistribution has been validated and characterized using a lipid vesicle model in a range of molar VSD concentrations (Zhang et al., 2017) [i.e., 1, 3, 6, and 9 μM] in a range of potassium gradients (Kang et al., 2019) [i.e., 25:1, 50:1, and 100:1 forming membrane potentials of −83, −102, and −120 mV, respectively] that create initial polarization to load VSD molecules into the vesicles. With a fixed initial membrane potential, fluorescence above 805 nm significantly increased with greater VSD concentration (Kang et al., 2020a). With a fixed VSD concentration, greater potassium gradients produced higher fluorescence intensities (Kang et al., 2019). Transient VSD responses were also characterized to define the temporal resolution using the lipid vesicle model (Kang et al., 2020a). 50% fluorescence intensity transients for depolarization events were achieved in 128.53 ms. In all, these studies indicated that generally fluorescence contrast upon membrane depolarization increases with higher VSD concentrations and higher potassium gradients, and provides a reasonable response time for neuroengineering studies. This paper focuses on further quantification of these trends in biological samples. The frame rates of the fluorescence imaging system were designed to reflect temporal VSD transition times. The established parameters were then applied to *in vivo* experiments to validate the feasibility of fluorescence imaging in detecting sufficient changes in signal due to stimulation.

### Optical Setup

A versatile wide-field fluorescence imaging system was built to evaluate the viability of IR-780 perchlorate, a near-IR VSD that operates by a transmembrane redistribution-based voltage sensing mechanism (576409, Sigma-Aldrich Corp., MO, United States), for monitoring neuron depolarization *in vivo*. The imaging setup, in the form of a benchtop system (Figure 1C), was designed based on the VSD excitation-emission characteristics (Figure 1B), following the same basic structure of the previously developed system for studying VSD delivery through the blood-brain barrier (BBB) (Pak et al., 2018). It was built with an 800-mW LED source centered at 780 nm accompanied by a condenser lens and an 800 nm cutoff shortpass filter to narrow the excitation bandwidth. An 805 nm cut-on dichroic mirror was used to separate the emission from excitation. Additional emission filtering was achieved with an 800 nm cut-on longpass filter. A zoom lens (Zoom 7000 Navitar Inc., NY, United States) was used to adjust focus for different target depths before the camera, an sCMOS Orca 4.0 (Hamamatsu Photonics K. K., Shizuoka, Japan). From this platform, two imaging configurations with different magnification scales were prepared to support both whole brain and cellular-level investigations (Figure 1C): (a) a large field-of-view (FOV) configuration that allows monitoring of an entire *ex vivo* brain slice or an *in vivo* mouse brain at once and (b) a microscopic configuration with greater magnification through the addition of an achromatic doublet that achieved cellular resolution for *in vitro* experiments.

### Experimental Setup and Design

**FIGURE 2:**
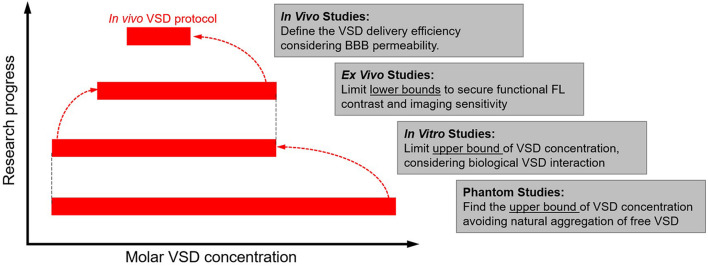
Experimental design flow chart to establish target VSD tissue concentrations that maximize fluorescence percentage change observable due to neuronal depolarization in rodents.

Regarding *ex vivo* sample setups, two specialized configurations were designed (Figure 1D): (1) a well plate configuration as the default setup and (2) a “bath” configuration was designed to reject confounding factors from the well plate experiments. The bath setup consists of a large central basin where brain slices were placed and two smaller basins for input and drainage of solution, enabling constant flow of new oxygenated medium over brain slices for improved cellular health during the course of the experiment. Figure 2 summarizes our progressive experimental approach for selection of optimal VSD concentration ranges, starting with phantom and *in vitro* characterizations that informed *ex vivo* and *in vivo* studies. Intravenous injection concentrations that targeted the maximum concentrations found in *in vitro* experiments were approximated for application in *ex vivo* and *in vivo* studies. These calculations are detailed in section “Estimation of tissue VSD concentrations *in vivo*.” taking into account dilution by the blood volume and partial permeability of the BBB. *The animal studies were reviewed and approved by the Johns Hopkins Animal Care and Use Committee (ACUC).*

#### Upper-Bound Voltage-Sensitive Dye Concentration to Maximize Fluorescence Sensitivity

The concentration-dependent aggregation of free VSD molecules was first investigated in tubing phantoms. Aggregation of VSD molecules in the extracellular matrix was expected in practical *in vivo* circumstances, leading to lower VSD signal sensitivity and functional contrast. Thus, the aim was to determine the highest VSD concentration free from such natural aggregations triggered by a high molecular density of VSD molecules. For this, a phantom study was designed to test the dependency of the fluorescence signal strength on the molar concentration of VSD, which was observed in the range from 0 μM (saline control) to 1,000 μM. Tubes filled with these VSD solutions were each placed in a consistent fixed position with sample configuration (a) in Figure 1C. The fluorescence signal intensity within the tubes was measured at each concentration and the molar concentration giving the highest fluorescence intensity was used as a reference upper bound for the following studies.

To go a step further to include biological interactions of individual neurons with VSD molecules, *in vitro* neuronal cultures were also tested. Mouse primary cortical neuronal (PCN) cultures were prepared in a well plate at a density of 1.5 × 10^6^ cells per plate. 2 mL of cell culture medium were mixed with 100 μL of VSD solution to produce resultant molar concentrations ranging from 0 μM (saline control) to 50 μM. Note that the 50-μM upper bound was determined from the aggregation threshold concentration defined in the previous tubing experiment, at which natural concentration-dependent fluorescence quenching can be avoided. The PCN cultures were then stained for 30 min for uniformity and to allow equilibration. Afterward, each culture was imaged by the fluorescence system with the cellular resolution configuration (b) in Figure 1C. The biological environment’s aggregation threshold was found by the trend in mean fluorescence intensity over images at different VSD concentrations and guided the following *ex vivo* and *in vivo* studies.

#### Lower-Bound Voltage-Sensitive Dye Concentration to Secure Functional Contrast

*Ex vivo* studies were conducted specifically to define the lowest bound of VSD concentration presenting functional contrast in fluorescence imaging, in which practical neural responses to external stimuli can be accurately modeled. All mouse studies reported here used CD1 mice, weighing ∼30 g (Charles Rivers Laboratory, Inc., MA, United States). Following our previous Regadenoson protocol (Pak et al., 2018), a catheter was placed in the tail vein of mice and VSD penetration through the BBB was facilitated by intravenous (IV) administration of Regadenoson prior to VSD injection. 75 or 150 μM VSD concentrations were used for IV injection. The mice were then sacrificed 2 – 3 min following VSD injection. The mice brains were harvested and then sectioned into 300-μm-thick slices without fixing or freezing. These whole brain slices were put into medium containing no VSD in the well plate sample configuration (1) from Figure 1D. The 4-min-long recordings were taken using imaging configuration (a) with potassium chloride (KCl) added at the 1-min mark, triggering cellular depolarization. For minimal disturbance during injection, KCl was injected through a tube that was secured at the side of each well, slightly submerged with no bubbles. Note that KCl concentrations up to 200 mM were used, consistent with previous studies that also employed up to a few hundred mM for cellular stimulation (Meier et al., 1988; Levitan et al., 1995; Shehata et al., 2012; Robertson et al., 2014; Rao et al., 2017).

The *ex vivo* studies were further elaborated to maximally reject confounding factors from the previous brain slice experiments. First of all, *ex vivo* brain slices were put in the “bath” configuration (2) in Figure 1D that constantly provides new supplies of oxygenated medium to increase the viability of cells. Secondly, coronal slices containing visual cortex only were used to reject regional variability in VSD delivery and responsiveness to stimulation. Thirdly, some mouse brain slices were stained directly with VSD medium ranging from 0.5 to 2.0 μM for 30 min for uniform staining and to set absolute VSD concentrations for the tissue samples. Stained slices were then placed in the bath containing medium with the corresponding VSD concentrations at which they were stained. Recordings were taken and KCl infusion was initiated from the 1-min mark via the input basin at concentrations of 50, 75, and 100 mM for varied stimulation strengths. Due to the gradual infusion of KCl into the main bath, recording time was extended to 8 min.

#### Estimation of Tissue Voltage-Sensitive Dye Concentrations *in vivo*

The same bath experiments and KCl procedures were performed on slices from mice that underwent systemic IV VSD and Regadenoson administration. Appropriate concentrations for IV VSD injections were chosen considering the molecular weight of the VSD (609.15 g/mol) and the total blood volume in a 30-g mouse of 1.76 mL (NC3Rs, 2021 Mouse: Decision tree for blood sampling | NC3Rs). Targeting a 10-μM VSD mean concentration in the bloodstream, as suggested in our *in vitro* study, 10.7 μg of solute should be injected. Limited by the total volume acceptable to inject into the mouse circulation (Animal care and use committee (2021) Species Specific Information: Mouse), it was determined that a 100-μL VSD injection volume was acceptable. With this volume, the VSD injection solution should be around 150 μM. Slices from these IV injected mice were first put into the bath with plain, no VSD medium flowing into the bath. For further evaluations, the slices from this same IV injected brain were stained with 2.0 μM VSD for 30 min for equilibration and tested with KCl as before. Comparisons of these VSD delivery methods (staining, intravenous injection, or both) to the tissue served the purpose of facilitating estimation of the VSD concentration delivered through the IV VSD and Regadenoson protocol developed previously.

Finally, to verify the relevance of these studies to the natural brain, *in vivo* studies were conducted. Each mouse, anesthetized with a 50:1 ketamine:xylazine solution, underwent a craniotomy surgery over the right brain hemisphere, spanning an area of about 8 mm x 10 mm. The craniotomy area was dried just before each recording but otherwise was covered with saline. Mice were again catheterized and were injected using our established protocol with Regadenoson and 100 μL of 150-μM VSD solution. Administration of KCl or saline solution was performed topically onto the exposed brain tissue in the craniotomy window. Fluorescence recording was performed for 5 min in total, and KCl or saline control was applied at the 1-min mark on the exposed brain region.

## Results

### Determination of Natural Voltage-Sensitive Dye Aggregation Thresholds in Phantom and *in vitro* Studies

**FIGURE 3:**
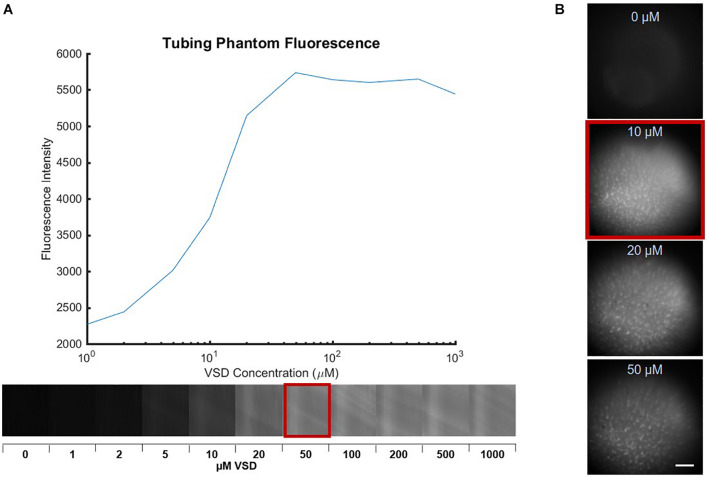
Fluorescence images of various VSD concentrations in **(A)** tube phantoms with the plot showing peak fluorescence at 50 μM, indicating the aggregation threshold in the absence biological cells and **(B)** PCN cultures with peak fluorescence at 10 μM, which further reduced the aggregation threshold. Scale bar = 650 μm.

Phantom experiments established the VSD concentration-dependent fluorescence in which increased fluorescence was observed with greater concentration up to a maximum fluorescence signal found at 50 μM, followed by decreased fluorescence signal for any higher concentrations (Figure 3A). This suggests that VSD concentrations under 50 μM produced negligible aggregation of free VSD molecules and thus, minimal fluorescence quenching was induced. Otherwise, at concentrations greater than 50 μM, VSD molecules began aggregating due to high population density, triggering fluorescence quenching. Therefore, we defined 50 μM as the maximum molar concentration of IR-780 perchlorate to avoid natural fluorescence quenching.

We further studied the biological interactions of our cyanine VSD molecules in *in vitro* PCN cultures in different VSD concentrations ranging from 0 to 50 μM. As shown in Figure 3B, the peak VSD fluorescence occurred at 10 μM, a concentration lower than that defined in the phantom study (50 μM). Higher molar concentrations at 20 and 50 μM showed decreased fluorescence *in vitro*. This indicated that the presence of the biological cell membrane altered the concentration-fluorescence relationship. This finding again validated the VSD mechanism as the cell acts like a small container encapsulated by a membrane that attracts positively charged VSD molecules inside with its negative resting membrane potential. The higher local VSD concentration intracellularly increased interactions between dye molecules and facilitated easier aggregation that led to fluorescence quenching. Therefore, it can be concluded that 10 μM is the effective molar concentration at which natural VSD aggregation is not triggered. Since our previous experiments using the lipid vesicle membrane model showed that higher VSD concentrations led to greater fractional changes in fluorescence (Zhang et al., 2017), it is also likely that a 10-μM VSD concentration would elicit the greatest fractional fluorescence change in neuronal cells.

### Determination of Minimum Voltage-Sensitive Dye Tissue Concentrations for Functional Contrast *ex vivo*

**FIGURE 4:**
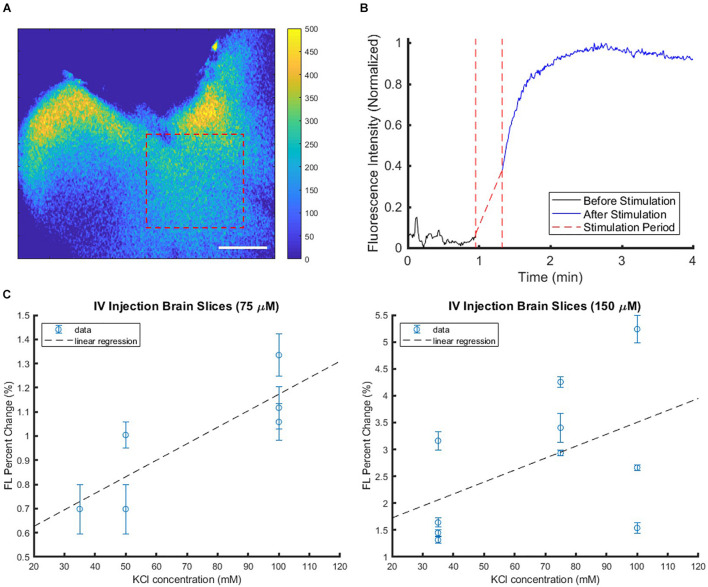
Sample brain slice with IV injected VSD in the well plate setup with KCl applied as a stimulant at the 1-min mark. **(A)** The subtraction colormap (image before stimulation subtracted from that after stimulation at 4 min) visualizes the stimulation-evoked changes that are also plotted in **(B)** which shows the increased fluorescence signal level post-stimulation. Scale bar = 2.5 mm. **(C)** Brain slice fluorescence responses were compiled at various KCl stimulation levels with VSD injection concentrations of 75 μM (left) or 150 μM (right) delivered intravenously. Each plot represents data collected from multiple slices from the same IV VSD-injected mouse and treated with different KCl concentrations. Each data point represents the mean fluorescence (FL) change of one slice, and the error bar indicates the variability within that slice, namely, how response strengths varied over different tissue ROIs within the slice.

The functional VSD contrast in response to membrane depolarization was tested through *ex vivo* brain slice studies. The addition of KCl, a depolarizing agent, acted as a stimulant. Figure 4A shows a representative fluorescence image of an *ex vivo* brain slice from a mouse injected with 150 μM VSD, and Figure 4B demonstrates corresponding intensity trace intensity over time from the region-of-interest (ROI) indicated by the dashed red rectangle in (A). The gradual increase in fluorescence to a peak was found following KCl administration and afterward, the elevated fluorescence level was sustained. Fluorescence responses, represented by the fractional change in intensity, at two VSD concentrations (75 and 150 μM) were plotted against KCl concentration, which ranged from 35 mM to 100 mM (Figure 4C). These results show generally an upward trend with increased fluorescence response correlated with greater stimulation and greater VSD injection concentration as indicated by the linear regression fits.

**FIGURE 5:**
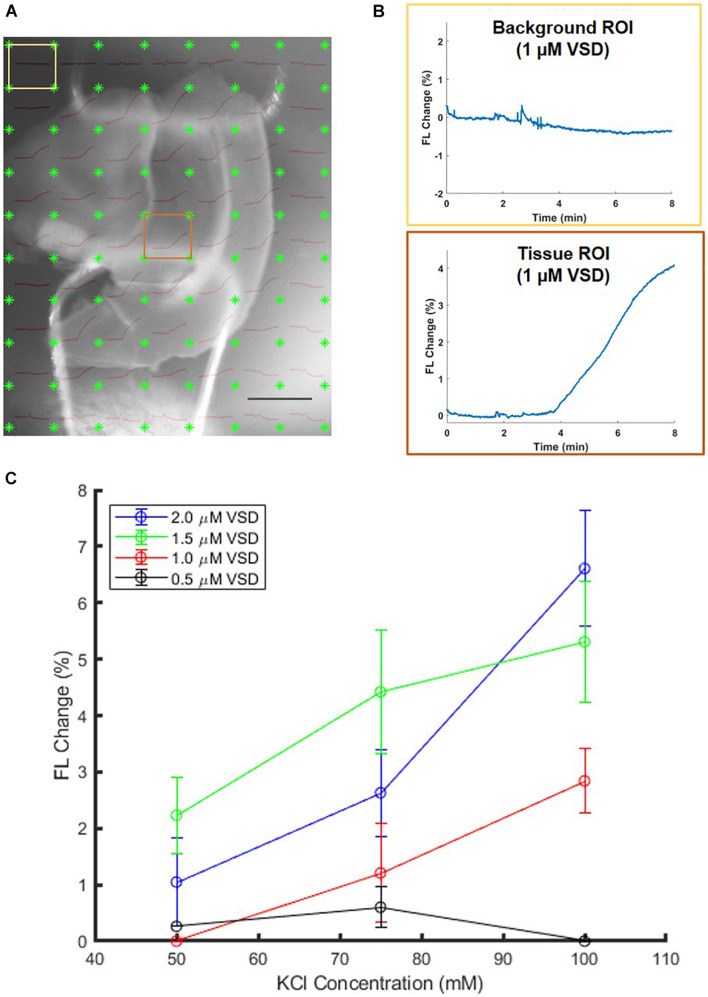
Sample 1-μM stained brain slice **(A)** anatomical image overlaid with fluorescence trace plots for each ROI. **(B)** The presence of differences in background and tissue ROI fluorescence change curves allows identification of minimum VSD concentration thresholds to achieve evoked responses. Scale bar = 2 mm. **(C)** Comparison of brain slices stained from 0.5 to 2.0 μM VSD at different stimulation strengths (KCl concentrations) indicates that with higher VSD concentration in the tissue, the difference in responses to different stimulation strengths is also greater.

To reject several confounding factors, the *ex vivo* experimental setup was further elaborated for clearer definition on the minimum VSD concentration that can provide functional contrast. *Ex vivo* brain slices that were directly soaked in VSD solutions were stimulated with KCl. If the tissue ROIs showed evoked responses while the background ROIs remained unchanged, it was determined that the VSD concentration under consideration was adequate for fluorescence contrast. The lowest VSD concentration for which the background and tissue ROIs showed significant differences in response was 1 μM, so this served as the lower VSD concentration threshold (example ROI differences in Figure 5A,B). VSD concentrations above this minimum threshold were further investigated.

When above the 1 μM threshold, it was found that the fractional change in fluorescence intensity increased with KCl concentration (Figure 5C) as expected and consistent with the aforementioned *ex vivo* study in the well plate configuration. The figure also indicates that higher VSD concentrations lead to greater sensitivity to differences in stimulation strength in general, namely the difference in responses due to low versus high KCl concentrations is greater than 5% for 2 μM VSD whereas for any concentration below 1.5 μM VSD, it is less than 3%. Thus, for maximal fluorescence responses and sensitivity to stimulation strength, it is optimal to have VSD concentrations in the upper part of the range between 1 and 10 μM in the tissue.

### Tissue Voltage-Sensitive Dye Concentration and Functional Contrast in Systemic Administration *in vivo*

**FIGURE 6:**
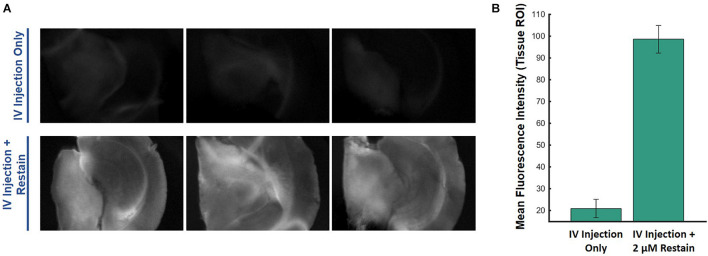
*Ex vivo* comparisons of **(A)** brain slices imaged from the same mouse injected intravenously with VSD at baseline (no stimulation) without (top) and with (bottom) re-staining using 2-μM medium, showing **(B)** significantly greater fluorescence signal with the re-staining process, which indicates that IV VSD injection delivered less than 2 μM of VSD to the visual cortex tissue.

Another experiment was conducted to estimate the VSD concentrations that reached the brain tissue when VSD was delivered through our IV injection protocol. The VSD fluorescence for *ex vivo* slices that originally received VSD through IV injection were compared to those that had IV VSD delivered in addition to direct re-staining for a 30-min equilibration period with 2 μM VSD medium solution (Figure 6). In the resting state, the directly re-stained slices appeared to have a greater VSD fluorescence intensity than the original slices with VSD delivered through intravenous means only, suggesting that IV VSD administration practically yields less than 2 μM local VSD concentrations in the visual cortex tissue. In comparing VSD delivery through IV injection to direct staining (Figure 4C versus Figure 5C, respectively), IV injection produced functional responses, indicated by fluorescence fractional changes, comparable to slices stained at 1.0 – 1.5 μM. Therefore, we concluded that the IV VSD delivery method used in this study provided 1 – 2 μM of mean VSD concentration to the brain tissue.

**FIGURE 7:**
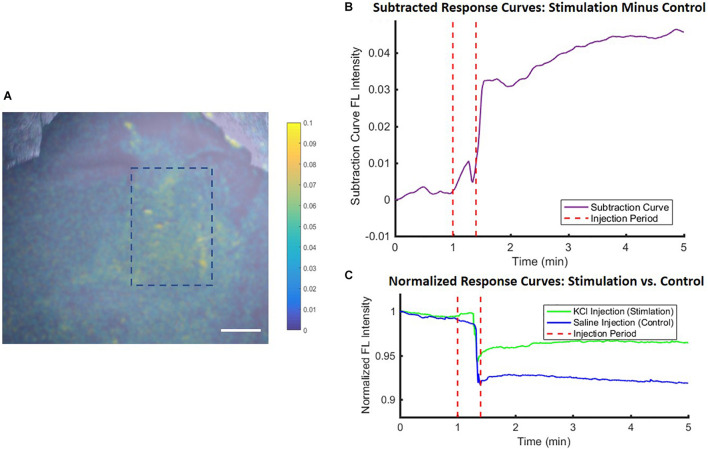
*In vivo* experimental proof-of-concept results showed similar trends to *ex vivo* studies. Differences between stimulation (KCl) and control (saline) conditions were used to account for injection liquid scattering **(A)** in a fluorescence image for anatomical mapping overlaid with a colormap representing the difference between the conditions and quantified in **(B)** the subtracted time course fluorescence traces, with **(C)** showing the individual KCl and saline normalized response curves, all averaged over an ROI within the stimulation region [blue dashed rectangle in **(A)**]. Scale bar = 4 mm.

Finally, the use of the VSD *in vivo* was also tested for proof-of-concept and to verify that the *ex vivo* studies reflect the same trends as those *in vivo*. Figure 7 shows the noticeable differences between the saline control and KCl stimulation groups, reflected by the subtraction of these response curves in (B). In both groups, a topical administration of exogenous liquid (saline or KCl) appears to show a sudden decrease in signal due, in part, to liquid scattering in the light pathway. However, once KCl is applied, the stimulation group indicated a gradual fluorescence increase which was negligible in the case of saline injections. This gradual increasing trend delayed slightly from the injection time was the same trend seen in the *ex vivo* studies, suggesting the direct applicability of *ex vivo* experiments. Thus, we concluded that local VSD concentrations delivered to brain tissue *in vivo* via our IV injection protocol were above 1 μM which was defined as the threshold to see the functional contrast in the *ex vivo* experiments aforementioned.

## Discussion and Conclusion

We have demonstrated the optimization of a functional fluorescence VSD imaging protocol through a series of phantom, *in vitro, ex vivo*, and *in vivo* studies to effectuate our near-IR VSD, IR-780 perchlorate, in rodents. The phantom study yielded the highest VSD fluorescence at 50 μM, which determined the threshold concentration for avoiding natural VSD aggregation and fluorescence quenching. In the following *in vitro* study, we found that the neuronal attraction of the VSD into the intracellular space lowered the effective VSD quenching threshold to 10 μM. The proceeding *ex vivo* studies revealed the positive correlation between VSD concentration and stimulation intensity at VSD concentrations higher than 1 μM. *In vivo* experiments designed to have VSD tissue concentrations in the range of 1 – 10 μM, following the guidelines established by our *in vitro* and *ex vivo* studies, showed functional fluorescence contrast upon depolarization triggered by topical KCl application. Therefore, the extensive translation from bench to *in vivo* studies validates our *in vivo* protocol for fluorescence VSD imaging in rodents.

Previously, other groups have successfully monitored neuronal depolarizations optically with high spatiotemporal resolution by way of RH and ANEPS VSDs that use different voltage-sensing mechanisms of action (Orbach et al., 1985; Shoham et al., 1999; Grinvald and Hildesheim, 2004; Carlson and Coulter, 2008; Ichi Nixima et al., 2017). These studies achieved fractional fluorescence changes of less than 1% whereas our *ex vivo* results show fractional changes up to 6-8%. However, it should be noted that no direct comparisons of data are possible as our experimental conditions did not match, such as our choices of VSD, stimulation methods and strengths, and sample types (*ex vivo* brain slices). The RH and ANEPS VSDs operate in the 600 nm wavelength range and in the visible range, respectively, so any live animal studies required craniotomy procedures. In contrast, our selection of VSD pushed farther into the near-infrared region to achieve greater penetration depth that facilitated through-skull imaging. This in addition to our studies that showed success in intravenous application of VSD as opposed to topical application used in other studies helped to achieve our ultimate goal of developing neuronal depolarization imaging methods that can be applied minimally invasively. Furthermore, we have conducted separate photoacoustic studies (Zhang et al., 2017; Kang et al., 2019, 2020b) with this VSD that demonstrate the complementary nature of fluorescence and photoacoustic signals that can be developed together to capture both the radiative and non-radiative processes simultaneously.

Despite the outcomes providing quantitative guidelines effectuating the fluorescence VSD imaging approach, there are still several confounding factors to be addressed in our continual studies. In the first *ex vivo* studies done in a well plate, mice received VSD through IV injection. These slices showed high variability in functional VSD fluorescence contrast within and between slices obtained over an entire mouse brain. This could be the result of spatial and subject variabilities in VSD delivery efficiency and/or responsiveness to stimulation. Even though our later studies suggested that tissue from the visual cortex received 1 – 2 μM of local VSD concentration, this cannot be generalized to other regions and neural circuitries, which were not investigated due to limited availability of mouse brains. Therefore, we will perform more detailed further studies, investigating individual VSD delivery efficiencies at distinct brain regions in the future.

In *ex vivo* experiments, the viability of cells may not be consistent over time in the well plate configuration, leading to degradation in VSD fluorescence response. Accordingly, the “bath” configuration was designed to address these concerns by supplying a constant flow of oxygenated medium to brain slices for which only the visual cortex was extracted. A direct VSD staining scheme was used to control local VSD concentrations in direct contact with tissue. On the other hand, with brain slices taken from IV injected mice in which the local tissue VSD concentration was unknown, medium without VSD was used in the bath. This was done to investigate the worst-case scenario or least response and to minimize changes to the VSD distribution. However, the bath configuration may introduce another confounding factor through flushing out VSD from these brain slices due to concentration gradients because of the use of plain medium. In particular, this effect may have been compounded in the “bath” setup due to the constant flow of new medium with 0 μM VSD over the slices. This factor was validated with additional *ex vivo* studies comparing well plate and bath configurations with the mice injected with the same 100 μL of 150-μM VSD solution. The fractional VSD fluorescence contrast was shown in the well plate setup upon KCl administration (Figure 4), but negligible contrast was found in the bath configuration. Therefore, this necessitates more attention and further investigation to consolidate the quantitative guidelines about the required VSD concentrations for IV injection to achieve the optimal VSD concentration in the brain tissue ROI.

As mentioned in the methods, injection concentrations and volumes were calculated based on the molecular weight of the VSD, total mouse blood volume, and limitations in total injectable volume. However, such an approach does not account for the further loss of local VSD concentration by partial efficiency in transferring the VSD from the blood vessels to the tissue through the BBB. BBB crossing efficiency could be expected to be below 20% (Gao et al., 2014), leading to a possible VSD tissue concentration around 1.7 μM. Since the first *ex vivo* studies in the well plate demonstrated stimulation-evoked responses, a range of 0.5 to 2.0 μM VSD concentrations was investigated to determine the minimum feasible VSD concentration for fluorescence stimulation response monitoring. In order to achieve a greater VSD tissue concentration for increased fluorescence fractional change contrast, multiple VSD injections may be considered in future studies.

In *in vivo* investigations, as mentioned above, there is still ambiguity remaining in determining and measuring the local VSD concentration delivered to the brain tissue from original VSD concentration injected, which could also vary between brain regions. In our study, local VSD concentrations in the visual cortex after IV VSD injection were estimated to be 1 – 2 μM from *ex vivo* experiments, suggesting a VSD delivery efficiency to the brain ranging from 12 to 24%. However, it is still inconclusive with the current study and the confounding factors aforementioned if the *in vivo* protocol achieved the maximum VSD fluorescence contrast. We plan to perform more controlled experiments at different stimulation strengths validating the quantitative efficiencies. This additional step would establish a guideline for optimal IV VSD injection to have closer to 10 μM of local VSD concentration which would maximize VSD fluorescence while avoiding extracellular VSD aggregation, as suggested in the *in vitro* study.

The use of KCl as a depolarizing agent has obvious limitations in spatial uniformity in samples immersed in medium and in the exposed brain *in vivo*. Nevertheless, other studies have also used KCl as a neuronal stimulation agent. Teichert, RW et al. used KCl to identify mouse lumbar dorsal root ganglion (DRG) neuronal cell subtypes in cell cultures in various receptor agonist challenges, presenting up to 200% fractional contrast changes with 100 mM KCl stimulation (Teichert et al., 2012). Chen, Y and Huang, LM also demonstrated detection of fluorescence changes of about 69% upon 80 mM KCl stimulation of DRG neuron cell cultures (Chen and Huang, 2017). Although values cannot be directly compared to our results as previously mentioned due to different experimental conditions and experimental setups, these studies show the same trends as our results, namely significant fluorescence elevation with application of similar KCl concentrations.

Another concern is that KCl concentrations above 50-90 mM may lead to neuronal injury or eventual death of cells (Ramnath et al., 1992; Takahashi et al., 1995) although these concentrations have been used in other neuronal stimulation experiments (Rienecker et al., 2020). Moreover, the topical KCl application *in vivo* produced diffuse reflection within the fluorescence imaging FOV, which hampered image analysis. To better estimate how much VSD was delivered using the stimulation strength dependent fluorescence response, it may be better to utilize physiological or electrical stimulation *in vivo* in future studies. Along with this, we plan to perform more extensive characterizations at various voltage transients and to provide direct correlates of voltage to fluorescence emissions. We will further secure statistical rigor in *in vitro*, *ex vivo* and *in vivo* experiments with better controlled and comprehensive experiments.

We will also perform more profound investigations to characterize the VSD properties in various scenarios, having various ranges of negative and positive voltage transients. Our previous studies only presented basic VSD characterizations in the voltage transients from −120 mV to 0 mV in lipid vesicle model. We will include more variant cases for complete characterization of VSD contrast and transient coefficients. Also, the degree of variability of the VSD mechanism due to temperature or pH changes was not tested in the current scope of our study. We accordingly plan to further characterize the effects on fluorescence intensity and molecular aggregation under such variations.

Overall, we have presented methods for optical imaging of neuronal activity that are minimally invasive in nature, facilitating translation into clinic. Together with our previous investigations on dye delivery to the brain tissue through the BBB, we have developed optimized protocols for administration of near-infrared VSD IR-780 perchlorate intravenously and established target VSD tissue concentrations for optimal fluorescence contrast in order to monitor neuronal depolarizations. Fluorescence imaging penetration depth benefited from the near-infrared excitation and emission spectra of our VSD, allowing invasive surgical procedures to be avoided and facilitating functional through-skull imaging with contrast maximized by application of VSD tissue concentrations of 1 – 10 μM. An integration with photoacoustic imaging, as investigated in our group’s previous studies, holds the potential to produce synergetic advantages in more reliable functional contrast and multi-modal contrast to enable novel neuroengineering research in the future.

## Data Availability Statement

The raw data supporting the conclusions of this article will be made available by the authors, without undue reservation.

## Ethics Statement

The animal study was reviewed and approved by Johns Hopkins Animal Care and Use Committee (ACUC).

## Author Contributions

JUK and EB conceived the idea of near-infrared voltage sensitive dye imaging. RP designed and built the optical setup, developed surgical protocols and custom mounts for animal procedures and imaging, developed analysis pipelines, and wrote the initial manuscript draft. RP and JK co-designed the experimental progression and acquired data. RP, JK, and JUK evaluated results and discussed improvements. All authors reviewed the manuscript.

## Conflict of Interest

The authors declare that the research was conducted in the absence of any commercial or financial relationships that could be construed as a potential conflict of interest.

## Publisher’s Note

All claims expressed in this article are solely those of the authors and do not necessarily represent those of their affiliated organizations, or those of the publisher, the editors and the reviewers. Any product that may be evaluated in this article, or claim that may be made by its manufacturer, is not guaranteed or endorsed by the publisher.
